# Perceptions of key stakeholders on taxes on tobacco and alcohol products in Nepal

**DOI:** 10.1136/bmjgh-2023-012040

**Published:** 2023-10-09

**Authors:** Yubraj Acharya, Vipassana Karmacharya, Uttam Paudel, Supriya Joshi, Ramesh Ghimire, Shiva Raj Adhikari

**Affiliations:** 1 Department of Health Policy and Administration, Pennsylvania State University, State College, Pennsylvania, USA; 2 Independent Researcher, Kathmandu, Nepal; 3 Chulalongkorn University, Bangkok, Thailand; 4 Pennsylvania State University, University Park, Pennsylvania, USA; 5 Central Department of Economics, Tribhuvan University, Kathmandu, Nepal; 6 Nepal Health Economics Association, Kathmandu, Nepal

**Keywords:** health policy

## Abstract

**Background:**

Non-communicable diseases (NCDs) are on the rise in Nepal. Consumption of alcohol and tobacco products remains high. Taxes on these products are significantly below the rate recommended by the WHO. In an effort to understand the reasons behind the slow progress towards the adoption of higher health taxes to curb NCDs, we documented the perceptions of key stakeholders on health taxes, including perceived barriers and facilitators to adopting higher health taxes.

**Methods:**

We conducted 45 in-depth interviews with individuals comprising government officials; producers, wholesale distributors and sellers of alcohol and tobacco products; and consumers and representatives from civil society organisations. We conducted a thematic analysis of the resulting data.

**Results:**

Respondents from alcohol and tobacco industries are not supportive of higher health taxes. They argued that higher taxes can increase illicit trade and worsen inequality. Strikingly, several government officials shared the industries’ concerns, arguing that health taxes have limited potential to reduce consumption of alcohol and tobacco products to help curb NCDs. In terms of barriers to adoption of higher health taxes, several local government representatives opined that close ties between industries and politicians at the federal level is a major hindrance.

**Conclusions:**

In order to adopt higher health taxes, the government will need to counter the false narrative pushed by alcohol and tobacco industries on the negative economic effects of such taxes. Health taxes earmarked for NCDs need to reflect the amount of revenue raised, reoriented towards prevention efforts and communicated clearly to the public.

**WHAT IS ALREADY KNOWN ON THIS TOPIC**

- Health taxes have the potential to reduce the consumption of alcohol and tobacco products and help curb non-communicable diseases.
- In Nepal, health taxes are below WHO-recommended rates and among the lowest in South Asia.

**WHAT THIS STUDY ADDS**

- This study is among the first efforts to document the perceptions of key stakeholders on the use of taxes on alcohol and tobacco products in Nepal.
- Support for higher taxes on alcohol and tobacco products is limited, with evidence of industries employing various tactics to prevent tax increase and to sustain public support.

**HOW THIS STUDY MIGHT AFFECT RESEARCH, PRACTICE OR POLICY**

- The study’s findings present advocates with several areas for action, in particular the need to publicise industries’ effort to undermine taxes and to general local evidence on economic effects of health taxes.

## Introduction

Non-communicable diseases (NCDs) are on the rise globally. They are responsible for more than 70% of all deaths, three-quarters of them in low-income and middle-income countries (LMICs).^1^ Health taxes can be integral to curbing the rising incidence of NCDs, as higher taxes can potentially reduce the consumption of harmful products while raising revenues to fund efforts to tackle NCDs.^2^ Health taxes can also contribute to poverty alleviation because they reduce medical expenditure and add years of productive life.^3 4^

In Nepal, the number of deaths caused by NCDs is rising.^5 6^ In 2016, NCD-related premature deaths accounted for 66% of all deaths and contributed to 51% of total disability-adjusted life-years.^5 7^ Among the NCDs, the highest proportion of premature deaths are attributable to cardiovascular diseases (30%), followed by chronic respiratory diseases (10%), and cancers (9%).^5^ Most NCDs cause death at an earlier age in Nepal than in high-income countries.^7^

The consumption of tobacco and alcohol products, which is a prominent risk factor for many NCDs,^8^ is high and rising. For example, the prevalence of tobacco consumption was 31.7% in 2020.^9^ An estimated 37 500 deaths (19.4% of all deaths) were attributed to tobacco-related diseases in 2019, up from 10.5% of all deaths in 1990.^10 11^ Alcohol consumption is similarly high; in 2019, 23.9% of all adults reported that they had consumed alcohol in the preceding 12 months and 11.7% of men reported that they drank daily or almost daily.^12^

The government of Nepal has formulated a number of policies on the production, marking and consumption of tobacco (table 1). Nepal has been a member of the WHO Framework Convention on Tobacco Control (WHO FCTC) since 2007. The Tobacco Product Control and Regulatory Act 2011 is the primary law governing tobacco control.^13^ Recently, the government has developed the FCTC Strategy 2030,^14^ the provisions on which are aligned with previous regulations, including the Tobacco Product Control and Regulatory Regulations (2011) and the associated directives.^15^

**Table 1:** Legislations on tobacco and alcohol products in Nepal

Policy, act, regulation, directive	Date of enactment	Objective, key provision	Provision(s) related to health taxes
Hotel Management and Sale and Distribution of Liquors (Control) Act	1966 (amended in 2010)	It restricted the sale and distribution of alcohols in hotels and set time restrictions on consumption.	None
Liquor Act (1974)	1974 (amended in 2000)	To control the production, sales and distribution of alcohol. It required those who were involved or wanted to get involved in the production, sales and distribution of alcohol products to obtain a licence from the government first.	None
Liquor Regulations (1976)	1976 (amended in 2008 and 2017)	To clarify the procedures for obtaining and renewing the license to produce, sell or distribute alcohol.	None
National Alcohol Regulation and Control Policy (2017)	2017	To decrease the availability and accessibility of alcohol products.	
National Anti-Tobacco Programme	1993	To reduce tobacco product consumption which would decrease morbidity and mortality related to tobacco use.	Makes provisions for excise tax on tobacco products to generate revenue and to discourage consumption.
Health Tax (Smoking and Liquor Charge) Fund Rules, 2051 (1994)	1994	It mandated the establishment of Health Tax Fund to collect and mobilise health taxes. Most of the revenues were to be allocated to the Bharatpur Cancer Hospital	
Tobacco Product Control and Regulatory Act 2011	2011	To reduce, control, and regulate the import, production, sale, distribution, and consumption of tobacco products.	Mandates to establish a Health Tax Fund to utilise the health taxes collected.
Directives for Printing and Labeling of Warning Message and Picture 2011	2011 (Amended in 2014)	To provide the details of printing and labelling of warning message and picture on the wrappers of commercial tobacco products.	None
Tobacco product Control and Regulatory Regulations (2012)	2012	To reduce, control, and regulate the import, production, sale, distribution, and consumption of tobacco products.	Mandates to establish a Health Tax Fund to use the health taxes collected.
National Tobacco Control Strategic Plan (2013–2016)	2013	To reduce mortality and morbidity attributable to tobacco consumption.	The plan aimed to increase the tax component of the price of tobacco products from 45% in 2013 to 66% by 2016.
Tobacco product Control and Regulatory Directives (2014)	2014	To reduce, control, and regulate the import, production, sale, distribution, and consumption of tobacco products.	Not applicable
The Public Health Service Act (2018)	2018	Article 45 banned the advertisement of alcohol, cigarette and tobacco products that has adverse effect on human health.	
Framework Convention on Tobacco Control 2030 Strategy: Nepal	2018	To control tobacco consumption by introducing both the tax and non-tax control measures.	Article 6 of the strategy aims to raise tobacco taxes.

The government has also adopted a range of measures to reduce harmful consumption of alcohol, starting with the formulation of Liquor Act (1974), which regulated the production, sales and distribution of alcohol products by issuing licenses to producers, distributors and retailers.^16^ Following the Act, Liquor Regulation 1976 was introduced, followed by the Health Tax (Smoking and Liquor Charge) Fund Rules 2051 (1994), which stipulated guidelines on the collection and mobilisation of health taxes.^17 18^ The most recent policy on alcohol envisions a ban on alcohol advertisement, promotion and sponsorship; reduction in the availability of alcohol through stricter licensing of distributors and reduced hours of operation of shops; increase in the age eligibility for drinking from 18 to 21 years; and a ban on the use of alcohol in government-sponsored events, educational institutions and sports activities.^19^

Beyond these efforts, as in many other LMICs,^20^ health taxes remain underutilised in Nepal. For example, in 2019, tobacco taxes were set at 15.5% of the retail price (excluding the value added tax (VAT)), representing one of the lowest in South Asia and far below the WHO standard of 70%.^9^ Two types of taxes are levied on these products: excise duty and health risk tax. (For imported products, there is an additional import tax. These products are also subject to a 13% VAT, which is a general rate for most products in Nepal.) In fiscal year (FY) 2022–2023 (Nepalese FY 2079–2080), the health risk tax was 30 paisa (25 US cents) and 60 paisa (50 US cents) per stick of bidi and cigarette, respectively, and Nepali Rupees (Rs) 60 (50 US cents) per kilogram of ready-to-chew items such as khaini, surti, gudhkha and paan masala.^21^ Excise duty on cigarettes is levied based on length, as shown in table 2. For example, in FY 2022–2023, excise duty for cigarettes without filter was Rs 710 (US$5.46) per metre.^21^ Likewise, excise duty on alcohol products are primarily set according to the concentration of alcohol and vary by volume, as shown in table 3, and remain low. Both excise duty and health risk task on alcohol and tobacco products are set during the announcement of the annual budget and collected by the national government.

**Table 2:** Taxes on tobacco products in FY 2022–2023

Product	Excise (Rs)	Unit
Not processed tobacco		
Not stemmed/stripped tobacco	130	Per kg
Stemmed/stripped tobacco	130	Per kg
Remnants of tobacco	130	Per kg
Cigar, cheroots, cigarettes and cigarillos, made from tobacco or tobacco substitutes		
Cigar, cheroots, and cigarillos	30	Per stick
Tobacco cigarette		
Without filter	710	Per metre
With filter		
Up to length of 70 mm	1635	Per metre
Up to length of 70 mm to 75 mm	2225	Per metre
Up to length of 75 mm to 85 mm	2880	Per metre
Length 85 mm and above	3965	Per metre
Other		
Readymade bidi	94	Per metre
All types of cigars	30	Per stick
Other types of cigarettes	30	Per stick
Water pipe tobacco (Tamakhu)	2000	Per kg
Processed tobacco for cigarettes and bidi	343	Per kg
Homogenised or reconstituted tobacco	460	Per kg
Chewing tobacco (Jarda, khaini, snuff, gutkha, nicotine-containing, paan spices and tobacco mixed products)		
Packaged tobacco mixed with lime for retail sale	460	Per kg
Cut tobacco or dust tobacco for wholesale	460	Per kg
Hookah flavour	1400	Per kg

Source: Government of Nepal. Arthik Ain Tatha Bidhayak 2079.

FY, fiscal year.

**Table 3:** Taxes on alcohol products

Product	Excise (Rs)	Unit
Sparkling/other wines containing		
Up to 12% alcohol	444	Per litre
12%–17% alcohol	444	Per litre
Above 17% alcohol	516	Per litre
Fermented alcoholic beverages		
Country beer (Chhyang)	43	Per litre
Champagne, Seri, Mid, Peri, Cider	516	Per litre
Liquors		
Raw materials including spirit for wine and brandy/whisky/rum/gin/geneva	228	Per litre
Brandy/whisky/rum/gin and geneva/vodka/liquors or cordials/other similar products containing:		
48.5% alcohol	1750	Per litre
42.5% alcohol	1306	Per litre
39.94% alcohol	1215	Per litre
34.23% alcohol	610	Per litre
28.53% alcohol	472	Per litre
17.12% alcohol	42	Per litre
Other alcohol products	1750	Per litre

Source: Government of Nepal. Arthik Ain Tatha Bidhayak 2079.

Overall, despite a strong global evidence on the effect of health taxes, low current levels of these taxes (relative to neighbouring countries as well as WHO recommendation), and the rapidly rising burden of NCDs, progress on raising taxes on alcohol and tobacco products has been slow. In an effort to understand the reasons for this slow progress, the primary objective of the current study was to document the perceptions of key stakeholders in Nepal on: (1) raising taxes on alcohol and tobacco products, (2) the taxes’ potential to help address NCDs, and (3) barriers to and opportunities for raising the taxes.

## Methods

To deliver the study’s objective, we conducted in-depth interviews with key stakeholders using a checklist of probe questions (online supplemental appendix 1). The interview guide was informed by the study objectives and published literature on the topic, and covered stakeholders’ perspectives on four key themes: higher taxation on alcohol and tobacco products, potential of health taxes for curbing NCDs, barriers and facilitators that may influence the adoption of health taxes in Nepal, and recommendations for an effective adoption of higher health taxes.

10.1136/bmjgh-2023-012040.supp1 Supplementary data

We sought to interview four types of stakeholders: (1) federal government officials, (2) provincial and local government officials, (3) producers (e.g., cigarette companies), wholesale suppliers, and retailers (shopkeepers) of alcohol and tobacco products and (4) consumers of alcohol and tobacco products, including civil society representatives. Note that for stakeholders in category (3) above, we asked questions pertaining to their product only (e.g., questions about tobacco were asked to producers, wholesale suppliers and retailers of tobacco, but not that of alcohol).

We first conducted in-depth interviews with federal and provincial government officials who were selected using purposive sampling and personal networks of the research team. We selected the government officials based on the research team’s knowledge of their potential role in designing or implementing taxes (see table 4). We then used stratified random sampling to identify one urban and one rural municipality in each province. In each of these municipalities, we identified participants using a mix of snowball and purposive sampling. We identified the first respondent in each municipality by visiting the municipality and requesting the officials (the mayor) for an interview. We then identified other respondents with their help, ensuring that respondents in each province encompassed multiple categories of stakeholders.

**Table 4:** Number of in-depth interviews, by province and category of stakeholders

Agency represented	Stakeholder category			
	A (n=6)	B (n=22)	C (n=12)	D (n=5)
**Federal level**				
Ministry of Health	x			
National Health, Education, Information Communication Centre	x			
National Planning Commission	x			
Nepal Revenue Advisory Board	x			
Ministry of Finance	x			
Inland Revenue Department	x			
Gorkha Lahari Cigarette			x	
Surya Nepal Cigarette			x	
**Province 1 (Koshi)**				
Revenue Officer		x		
Mayor		x		
Alcohol Producer			x	
Information Officer at a Hospital				x
**Province 2 (Madhesh)**				
Finance Mministry Rrepresentative		x		
Health Ministry Representative		x		
Ward Chairperson		x		
Mayor		x		
**Province 3 (Bagmati)**				
Finance Ministry Representative		x		
Health Ministry Representative		x		
Federation of Industry and Commerce				x
Municipality Health Officer		x		
Municipality Revenue Officer		x		
Social Development Ministry Representative		x		
Health worker				x
Ward Secretary		x		
Mayor		x		
**Province 4 (Gandaki)**				
Home and Small Industries Representative			x	
Chamber of Commerce Representative			x	
Deputy Mayor		x		
Ministry of Health and Population Representative			x	
Wholesale Supplier of Alcohol			x	
**Province 5 (Lumbini)**				
Home and Small Industries Representative			x	
Ministry of Economics Affair and Planning Representative		x		
Wholesale Supplier of Tobacco and Cigarette			x	
Ministry of Industries Representative		x		
Deputy Mayor		x		
**Province 6 (Karnali)**				
Health Officer				x
Home and Small Industries Representative			x	
Mayor		x		
Municipality Chief Executive Officer		x		
Wholesale Supplier of Alcohol			x	
**Province 7 (Sudur Pashchim)**				
Home and Small Industries Representative			x	
Mayor		x		
Ministry of Social Development Representative		x		
Supplier of Tobacco			x	
Consumer Protection Forum Representative				x
Ward Chairperson		x		

Note: The four stakeholder categories used in coding the responses are as follows. (A) Federal government official, (B) provincial and local government official, (C) producer, wholesale supplier or retailer of tobacco and alcohol products, (D) consumers and civil society representative.

We included participants at lower levels of the government in the study because, although taxes on alcohol and tobacco products are determined at the federal level, certain aspects of implementing tobacco-control and alcohol-control policies lie within the jurisdiction of provincial and local governments. For example, retailers selling alcohol products are required to obtain licence from, and pay registration and renewal fees to, local governments. Local governments also often run public awareness campaigns, for example on smoking cessation. Therefore, local government officers, such as mayors and chief of administration, may have insights on the challenges on implementing effective health taxes.

Data analysis was based on the iterative and cyclical process between literature, field data, synthesis and analysis, and interaction among the authors to agree on the content and interpretation.^22^ Recorded interview data were transcribed, translated to English and anonymised. Each respondent was given a three-digit code consisting of a letter pertaining to the profession and a two-digit number pertaining to an individual. We used A for federal government officials; B for provincial and local government officials, including health workers; C for producers, distributors and sellers of alcohol and tobacco products, and their umbrella organisations (collectively referred to as suppliers); and D for consumers and civil society representatives. We categorised the stakeholders in this manner based on our prior expectation of their perceptions on health taxes.

We conducted a thematic analysis that resulted from the literature on stakeholders’ perspectives on health taxes and potential barriers to raising and mobilising such taxes to curb NCDs in LMICs, and was consistent with our study objectives. To ensure reliability, SJ coded the dataset and YA randomly checked the codes for selected interviews. We used lump coding as opposed to line coding so as to understand the broader perceptions of health taxes. In the first coding cycle, we used the initial coding for the process of familiarisation and coding framework modification. Initial coding resulted in 28 codes and subcodes, including additional codes that emerged from the data. In the second phase, we used pattern coding to group the summaries into a smaller number of categories, themes and concepts.

Our analysis of the context, key actors and processes follows the Health Policy Analysis Triangle framework.^23^ The framework assumes that an understanding of policy should be informed by an analysis of policy context, content, process and actors, with actors at the centre of the triangle.^24^ We used NVivo software to organise and analyse the data.^25^

### Patient and public involvement

Patients and the public were not involved in the design, conduct, reporting or dissemination of our research.

## Results

Our final data consisted of responses obtained from 45 in-depth interviews. The list of key respondents is in table 4. In the overall sample, we had 6 central-level government officials, 22 provincial-level and local-level officials, 12 producers, wholesale suppliers, or retailers of tobacco and alcohol products, and 5 consumers or civil society representatives.

In this section, we present our results under four areas: (1) stakeholder views (including support for and against health taxes); (2) perceived barriers and opportunities for raising health taxes; (3) views on the use of health taxes to address the rising incidence of NCDs and (4) recommendations for an effective mobilisation of higher health taxes. Note that the codes next to the quotes refer to the stakeholder category. For example, ‘[C32]’ means that the comment was made by a producer, wholesale supplier, or a retailer of either alcohol or tobacco industries.

### Stakeholders’ views and support for and against health taxes

There is limited support among the stakeholders for health taxes in Nepal. The most prominent opposition to health taxes comes from producers of alcohol and tobacco products. One of the most commonly cited concern against higher taxes on these products was a possible rise in illicit trade (ie, products moving from India to Nepal illegally), as the following comment from a wholesale supplier of tobacco illustrates:

Taxing [in Nepal] just means more will come from India. Right now, it has been 6 months that the government has not allowed foreign alcohol to Nepal, but go to any dealer and you will find foreign alcohols easily. The same thing will happen. [C16]

Another concern cited was that health taxes would worsen inequality. One tobacco industry respondent said that ‘We are very honest and tell people that smoking is bad for health. But daily-wage labourers cannot work without smoking. They are addicted. When tax increases, these poor people have to pay more.’ [C18]

Industry respondents put forward a myriad of other arguments for why higher taxes should not be imposed on cigarettes and alcohol. They even questioned the validity of evidence presented by proponents of higher taxes, as the following comment from a cigarette company respondent shows:

During any budget debates, the government side and other anti-cigarettes always look at reports and studies of western countries and try to implement that here. But our attitude, education, culture, lifestyle is different, so it doesn’t make sense that they look at the western policies and try to impose that here in Nepal—this just further creates disparities and a rift from making policies to having the impact we intend. [C32]

Pointing to other factors that drive consumption of tobacco was a recurring theme among tobacco industry respondents, as the following comment illustrates:

We shouldn’t just look at one aspect and expect to understand the complex dynamics and interaction amongst the various factors that exist around cigarette consumption—just looking at the price increase through tax increase and consumption going down is invalid and inappropriate and misguided. [C21]

One supplier pointed to anecdotal evidence on the relative inelasticity of consumption of harmful products to price changes to support their argument on why health taxes would be ineffective. ‘During COVID-19, prices of cigarettes increased from Rs 5 (3 US cents) to Rs 8 (6 US cents). This did not affect (the) sale of cigarettes at all. People were willing to pay Rs 8 for the cigarettes for which they were paying only Rs 5.” [C15] Another supplier said, ‘These are not basic needs. People consume despite knowing the risks, so higher prices will not make them drink or smoke less.’ [C16]

Another supplier went as far as to suggest that alcohol and tobacco industries were discriminated against. They said, ‘We are judged and discriminated even though we pay more taxes. Why should we pay more tax than others?’ [C27]

Several industry representatives said that, instead of trying to raise taxes on alcohol and tobacco products that are already being regulated, the government should try to regulate low-end products that they said are potentially more harmful and often consumed by poorer individuals. As one alcohol industry respondent put it, ‘Branding home-produced alcohol can make it easier for the government to regulate them.’ [C38]

Strikingly, even the federal government officials seem to believe that reducing consumption of harmful products through higher taxes would affect the economy adversely, at least in the near term. One government official said, ‘Long term benefits would be higher. In the short term, it would cause an economic crisis in the country.’ [A13]

Many local government officials and consumers echoed the concerns expressed by suppliers, especially with regard to disproportionately higher adverse effects on the poor. For example, one local government official said that ‘price increase [would] induce low-income individuals to switch to lower quality products, further harming their health. Rich people, instead, can continue to afford the good-quality products.’ [B25]

### Views on the potential of health taxes for curbing NCDs

Many respondents understood the potential adverse effects of consuming alcohol and tobacco on health, and that ‘health hazards can be prevented by consuming less of these products.’ [B25]

However, nearly all respondents opined that reducing the consumption of alcohol and tobacco through health taxes alone—in a manner that would reduce NCDs—would be difficult. The reasons the respondents provided were as follows. First, substitutes are easily available. For example, ‘If price of cigarette increases because of taxes, individuals can switch to bidis, which are cheaper.’ [C30] Similarly, ‘if the price of rajanigandha [a tobacco product] increases, people can shift to bhola [another tobacco product].’ [B22]

Second, the respondents said that the consumption of alcohol and tobacco is often triggered and maintained by social stressors, often leading to addiction. ‘Because of addiction, people will consume cigarette and alcohol no matter what.’ [C27] They said that alternative measures, such as ‘banning the consumption of these products in certain spaces’ and providing ‘additional education on the risks of consumption’ [C15], would be needed to tackle the factors underlying persistent consumption.

Finally, several respondents—representing all stakeholder groups—pointed to culture as a reason for drinking and smoking. As one local government officials put it, ‘culture plays a major role in the consumption of alcohol. For example, in Newar communities, it is common to have alcohol in almost every auspicious occasion as sagun. In Tamang communities, when infants cry and the parents need to go to work, the infants are fed food and a small amount of alcohol to get them to sleep.’ [B29] A consumer said that ‘In western Nepal, women smoke bidi in groups during their afternoon break from household chores.’ [D36] The researchers encountered at least one case of an elementary school child carrying home-made alcohol to school for afternoon snack.

### Perceived barriers and opportunities for health taxes

Respondents in the study pointed out a number of potential barriers to raising health taxes. The following barriers were mentioned:

Influence of industries: Federal and local government officials identified resistance from powerful tobacco and alcohol industries as a major challenge to raising health taxes. They mentioned that intimate relations exist between the political parties and tobacco and alcohol industries and that political parties receive funds from the industries to finance their political activities. As one federal government official put it, ‘When a political leader says we are going to need election expenses, giving Rs. 10 crores [approximately, 77 000 US Dollars] at once is not a big deal for these industries.’ [A12]

Government officials from all levels mentioned that industries employ a myriad of techniques to influence policy-making, including under the pretext of corporate social responsibility (CSR). The techniques include supporting social causes (e.g., sporting events and relief efforts during natural disasters) and promoting their products at these events, and leveraging civil society leaders, parliamentarians, and lawyers. One respondent mentioned that tobacco and alcohol industries were not allowed to use money in CSR, but found ways to promote their products and create public support towards them nonetheless: ‘Tobacco and alcohol industries are not allowed to use money in CSR. It is against the policy. But they are using it. For example, during COVID, they helped in distributing ventilators when [local governments] were short in budget and promoted their products using company logos. They carried banners with their company’s name and logo when distributing relief materials during the earthquake, too.’ [A11]

Spreading misinformation and creating fear was also mentioned as a common tactic used by industries. One civil society representative said, ‘Industry people spread rumour that the economy would collapse without their industries and our country will be like Sri Lanka. They challenge the government and give examples of individuals who consume cigarettes and have lived long, implying that tobacco and alcohol are not harmful.’ [D36]

### Government’s capacity to implement health taxes

Many respondents—representing all stakeholder groups—expressed concerns about the government’s ability to implement higher taxes, even if the taxes were raised on paper. They pointed to a number of context-specific challenges. The first challenge was the government’s ability to communicate the changes in policy of any kind with relevant stakeholders. For example, one local government official said that there were guidelines stating that alcohol should be sold only between 6 and 9 pm, but ‘no alcohol business [knew] about it’ [B18] and stores were found to be open ‘when [they] went for monitoring at 2 pm in the afternoon. The shopkeeper did not know about the guidelines at all.’ [B18]

The second challenge was the government’s capacity to enforce laws, including penalising those who exploit loopholes. As one local official put it, ‘In Nepal, making laws and regulations is not a problem, but implementing them is a major challenge. Industries can easily trick the tax system. For example, if taxes on one product go up, they can introduce a product with a different name but with the same ingredient to avoid paying taxes.’ [B24]

A final implementation challenge that respondents identified relates to lack of clear mechanisms for coordination and communication between different levels of government in the new federal setup. For example, one federal government officials said, ‘How to regulate cigarette and gutka shops is already determined by the central government. However, enforcement of these guidelines is the prerogative of the local governments.’ [A14] One local official suggested that it would be more effective for the central government to regulate alcohol and tobacco markets—and not just set the excise duty on these products—as these ‘need to be regularly monitored’ and so that non-tax policies central to addressing NCDs are ‘similar in all places within the country.’ [B28]

### Cumbersome tax collection system

Suppliers of alcohol and tobacco products complained about the complex and cumbersome tax system. They pointed to the high number of the types of taxes and argued that the procedures for paying taxes were complex, including on tobacco and alcohol products. As one producer put it, ‘One of the reasons many people do not pay taxes is that they do not know where to go and what procedures to follow in order to pay the taxes.’ [C34]

A few suppliers said that the current tax system was unfair as it did not reward regular payers or penalise those who tried to avoid taxes. In one respondent’s words, ‘Those who pay taxes regularly should get rebates as rewards. Otherwise, there is no incentive for us to keep paying taxes when others who do not pay the taxes do not face any penalty.’ [C31]

The only opportunity for the adoption of higher health taxes that was mentioned by the respondents—of all stakeholder categories— were recent changes in the tax collection system, particularly digitisation and one-door policy for paying taxes, although these changes are not specific to taxes on alcohol and tobacco products. The respondents suggested expanding online system for paying taxes to rural areas and to all forms of taxes. The following three comments are illustrative of the respondents’ views on this area.

Many things have been digitized—I feel like it is heading in the right direction. It should be one door for paying tax—where people can easily pay tax so it is not complicated. [C35]

All our tax collection is done online and we have online transparency in taxes. [B34]

It would be easier if we could have this system in other rural places across all sectors (property tax, utility taxes etc) so that they wouldn’t have to physically go to pay taxes as well. [B18]

### Opposition from consumers

Given that enforcing higher health taxes can be viewed by opponents of such taxes as curtailing individual freedom, we asked the respondents if consumers of tobacco and alcohol products can be potential barriers. Consumers and government officials indicated that direct opposition from consumers is unlikely but pointed to opposition from political leaders for fear of losing votes. For example, one government official said that, unlike in the cases of hikes in petrol and diesel prices—a common occurrence in Nepal—‘There [would] not be any direct opposition to higher prices of harmful products.’ [A13] However, as one civil society representative indicated, ‘There might be resentment among the consumers, triggering an opposition from politicians who rely on these consumers for votes.’ [D15]

### Recommendations and the way forward

Respondents were asked what the government should do in order to be able to raise taxes on alcohol and tobacco products and make health taxes a more effective tool for curbing NCDs. A number of insights emerged, which warrant further research and analysis.

One federal government official emphasised that politicians and bureaucrats need to be secretive about potential increases in health taxes during the preparation of the annual budget (when tax rates are determined). The official said that ‘If the plans to raise health taxes are known beforehand, industries attempt to derail the efforts or hoard the products illegally to create artificial shortages’ [A11]. ‘While making the budget, we need to maintain certain secrecy.’ [A11]

Respondents provided several suggestions on the current earmarking of health taxes for NCDs. One local government official asked for greater transparency from the federal government. As they put it, ‘The government currently allocates 400 million rupees [308 000 US dollars] annually for NCD-related programmes, but on what basis? The figure should be transparent so consumers also feel ownership of the taxes.’ [B19] Several local officials and consumers suggested that the amount allocated to NCDs should be a fixed percentage of the revenue raised through health taxes—thus adjusted each year—and not a flat amount. That would enable consumers to see that a proportion of their taxes is being spent for their benefit, thus raising ownership of the taxes. A few local government officials suggested that health taxes should be collected *and* disbursed at the local level by local governments to ensure greater transparency. They further suggested that the funding should be used for ‘preventive care on NCDs, not on treatment’ [B22].

Several respondents pointed to the need to strengthen the enforcement of existing laws on alcohol and tobacco products. However, many of the suggestions the respondents provided on curbing NCDs related to non-tax interventions. Respondents were convinced that one of the factors driving the consumption of alcohol and tobacco products is the availability of these products. Suggestions on reducing access ranged from ‘setting specific time and place for purchase and consumption’ [B21] to ‘limiting the number of stores that can sell these products’ [D10], such as allowing only ‘5 authorised shops in one ward’. [D10, B21] Other suggestions included stricter screening of consumers for ‘age and pregnancy, restricting consumption in public spaces, and counselling and awareness programmes, including those targeted to individuals with addiction to drinking and smoking.’ [C30]

## Discussion

Using data collected through in-depth interviews among the key stakeholders on health taxes, we sought to understand perceptions of key stakeholders in Nepal on health taxes and barriers and facilitators to higher health taxes. Among the key findings, we found that industries are not supportive of higher taxes on alcohol and tobacco products and that the close link between politicians and these industries may be a significant barrier to the adoption of higher health taxes. Industry representatives expressed a myriad of concerns, many of which are either false or can be easily refuted using evidence from other countries. Some claims, such as the potential rise in illicit trade from India, seem ill informed as prices are currently lower than in India; if anything, if prices increase in Nepal due to higher taxes, illicit trade should fall. Strikingly, many government officials shared concerns similar to those of industry representatives. Stakeholders of all types pointed to the generally weak capacity of the government as another barrier to an effective adoption of higher health taxes. Regarding the role of health taxes to curb NCDs, most respondents argued that health taxes alone would be insufficient to reduce the consumption of harmful products and that additional complementary initiatives, such as smoking cessation programmes, would be needed.

These findings should be understood in light of a number of limitations. First, we relied on snowball sampling to identify respondents. Their views may not be representative of the overall population and are vulnerable to desirability bias. Second, we did not assess stakeholders’ understanding of health taxes nor did we provide them information from other countries on potential long-term effects of such taxes. Thus, their position may reflect the insufficient information they have and the tendency to prioritise immediate benefits and costs of health taxes on themselves. This may be true even for representatives from alcohol and tobacco industries. Further corroboration and clarification of our findings through a larger representative survey is the natural next step in this line of research.

The study’s findings present advocates with several opportunities. There exists a need to dispel misperceptions—many of them pushed by industries—that higher health taxes can be detrimental to the economy. Empirically, in many countries, reductions in tax revenue from decreased consumption have been found to be more than offset by the rise in revenue from those who continue to use these products, leading to an overall increase in revenue in response to tax.^26^ Higher taxes on tobacco have been found to have no net loss on jobs; they have been found to lead to modest job gains instead.^26^ This has been found to be true for alcohol as well.^27^ The gains of health benefits following health taxes tend to be progressive. For example, long-term medical costs have declined in Chile and Moldova following tax increases on tobacco products.^4 28^ Similar evidence from Nepal on health taxes’ potential economic effects will be needed to counter the industries’ arguments. Concurrently, the media, the civil society, and the public can help by publicising the efforts of the political lobby to undermine taxes, including industries’ efforts to influence the public in the pretext of CSR.

Relatedly, separate guidelines on mobilising health taxes are needed. These guidelines can clarify the breadth of items covered by health taxes, the roles and responsibilities of the three layers of the government, as well as how the revenue collected is to be used. Respondents suggested that the amount allocated to NCD efforts needs to be adjusted each year based on the revenues from health taxes that year and should be allocated to NCD prevention efforts. The need for greater transparency will need to be balanced with practicality, however. Currently, health taxes are collected as excise fees, health risk tax, or VAT. Separating the three, and parsing out the amount of revenues collected from different products, may be administratively costly. Nonetheless, as previous studies have pointed out, Nepal’s basis for taxation is confusing and can be simplified—for example, cigarettes are taxed based on their length rather than the number of packets produced which makes tax assessment difficult and avoidance easier.

In terms of allocations of revenues from health taxes to specific activities, there are a number of effective efforts from other countries that can be replicated. For example, Turkey has earmarked some of its tobacco tax revenues to help tobacco farmers shift to other crops.^24^ Youth smoking prevention and cessation programmes and Alcohol Anonymous-type efforts are some other examples of prevention programmes health tax revenues can finance. As a general recommendation, stress plays an important role in triggering the consumption of harmful products, as respondents in this study pointed out. Therefore, for health taxes to be effective in reducing consumption of harmful products and addressing the rising incidence of NCDs, the taxes need to be complemented with counselling programmes targeted to vulnerable groups (which again can be financed through health taxes), stricter enforcement of current ban in consumption in certain places, and additional education programmes on the risks of consumption targeted to youth in particular. Likewise, taxing all tobacco alcohol products equally can reduce the possibility of individuals switching to lower-quality and potentially more harmful products.

More generally, advocates of higher health taxes, such as the WHO, should continue to identify and support champions within the government and among the bureaucrats so they can be more vocal in exposing and countering industries’ conduct. Alcohol and tobacco industries working aggressively to prevent future policies using their resource advantage, including by presenting misleading economic arguments, is not new or unique to Nepal.^29^ However, with a strong overt support from the highest political leadership, significant reforms have been possible in other countries, such as the tobacco excise tax reform in the Philippines.^30^ Nepal’s own experience in other areas, such as the reform of the state-owned Agricultural Development Bank which we have documented previously, suggests that substantial reforms are possible if there is political will, even amidst controversies and seemingly challenging capacity constraints.^31^

### Conclusions

Several challenges exist in Nepal’s adoption of higher health taxes to curb NCDs. Chief among these challenges are lobbying by the industries against raising taxes and the government’s generally weak capacity on tax administration. Efforts to dispel misconceptions about the effect of higher taxes on the economy are needed. To increase public support for higher taxes, health taxes earmarked for NCDs need to reflect the amount of revenue raised, reoriented towards NCD prevention efforts and communicated clearly to the public.

## Data Availability

All data relevant to the study are included in the article or uploaded as online supplemental information.
