# Supplementary material for: Perceptions of key stakeholders on taxes on tobacco and alcohol products in Nepal
Source: BMJ Glob Health. 2023 Oct 9;8(Suppl 8):e012040. doi: 10.1136/bmjgh-2023-012040 (PMC10565236; doi:10.1136/bmjgh-2023-012040)
Supplement: Supplementary data [file bmjgh-2023-012040supp001.pdf]

Appendix 1. List of Questions Used in the In-depth Interviews

<u>Sub-topic: Stakeholder views (perceptions) on taxation for Tobacco, sugary products, alcohol and processed foods</u>

Section A: Effectiveness (Advantages and disadvantages of health tax)

1. In your opinion, what effects would be on the purchase and consumption of tobacco, sugary products, alcohol and processed foods due to incremental health tax rate? (Probe for local alcohol brand, imported foods, doubts on effects)

2. What effects would you feel on the price of tobacco, sugary products, alcohol (including local) and processed foods (including imported) due to the increase in health tax rate? (Probe for sin tax vs. retail prices)

3. What major sectors would be affected with largest effects of rising prices of Tobacco, sugary products, alcohol and processed foods due to the increase in health tax rate? (Probe for sin tax vs. multiple effects) [Signaling effects]

4. What kind of health outcomes can be expected after applying the incremental health tax rate? (Probe for improvement, doubts, effects, collaboration with other strategies etc.)

5. Do you feel any support of effectiveness based scientific evidence to apply incremental health tax in Nepal? (Probe for need and no need of the evidence)

6. In your thought, what strategy in productions of tobacco, sugary products, alcohol and processed foods should be applied by the industry if the tax rate is increased? (Probe for reformulation, alternatives, production strategy, shift to alternative healthy products or no concern to shift)

Section B. Appropriateness of health tax as a target for interventions

7. Do you think the health tax can be appropriate target to reduce the prevalence of non-communicable diseases (NCDs)? [Appropriateness]

8. Do you think excessive focus on incremental health tax possibly cause on increasing NCDs in Nepal? (Probe for targeting health tax – fair/unfair for industries and consumers)

9. In your opinion, how can the incremental health taxation system be more appropriate to reduce NCDs caused by these products? (Probe for government intervention, preference for voluntary solutions, individual freedom, coverage, universal prevention strategy for appropriateness/not appropriate)

10. Do you think the incremental health tax is appropriate as necessary or sufficient to reduce consumption and production of these products to reduce NCDs?

11. How will incremental health tax rate respond to the financial equality? (Probe for regressive or progressive effects)

12. How will incremental health tax rate respond to the health and socioeconomic equality? (Probe for regressive or progressive effects in terms of health benefits)

Acharya Y, *et al. BMJ Glob Health* 2023; 8:e012040. doi: 10.1136/bmjgh-2023-012040

13. In your thought, what economic consequences in health system will be created through the incremental health tax rate? (Probe on cost savings, workforce etc.)

Section C: Economic Consequences

14. What consequences will be seemed in industry’s profit and employment due to the insertion of incremental health tax?

15. In your opinion, what effects can be expected through generating tax revenues from incremental health tax? (Probe on economic consequences)

16. What would be the cross-border issue of incremental health tax? (Probe with NCD prevalence)

17. Do you think, health tax can change the market forces (distortion) in the economy? If yes how?

<u>Sub-topic: Barriers and facilitators that (according to stakeholders) may influence the adoption of health tax in Nepal</u>

Section E: NCDs and voluntary industry actions, Political Context and Lobbying

18. What can be the major barriers and facilitators to influence the adoption of health tax in Nepal in terms of NCDs and voluntary industries’ actions?

19. What can be the major barriers and facilitators to influence the adoption of health tax in Nepal in terms of political context? (Probe for interest, election goal, ministerial agenda, budget, gov. change etc)

20. What can be the major barriers and facilitators to influence the adoption of health tax in Nepal in terms of lobbying? (Probe for lobby of the health sector and societal organizations, political power and resources of health sectors, political power and lobby within the industries)

Section F: Public Acceptability, International Context, Legal Framework and Technical Feasibility

21. What barriers and facilitators would be experienced in terms of public acceptability if incremental health tax is adopted in Nepal? (Probe for public opinion, culture heritage, other social issues)

22. What barriers and facilitators would be experienced in terms of international context if incremental health tax is adopted in Nepal? (Probe for tax in other countries, WHO recommendation, so on)

23. What barriers and facilitators would be experienced in terms of legal framework if incremental health tax is adopted in Nepal? (Probe for available legal basis and reforms)

24. Do you think, is it technically feasible to adopt health tax in Nepal? What facilitators and barriers will be experienced? (Probe for understanding health tax, administrative needs and

management, future influencing factors)

<u>Sub-topic: An overview of stakeholder recommendations for the design of health tax if introduced in the Nepalese context</u>

Section G: Definition of health tax, Health tax rate, Use of revenue, Communication policy and Monitoring

25. How do you like to define health tax in the context of Nepal?

26. Which health tax type/base/rate will be the best practice to be adopted in Nepal? (Less/more than 35%, flat tax, multiple tiered tax)

27. How do you like to recommend in the best use of revenues from health tax in Nepal, so that NCDs prevalence can be addressed?

28. What would be the best communication policy and to whom the tax policy is to be informed to make it popular among producers and consumers in reducing NCDs?

29. What will be your recommendation to monitor the tax intervention for NCDs reduction? Does this policy need to be combined/integrated to other tax policies?
